# Effect of agricultural practices on terrestrial isopods: a review

**DOI:** 10.3897/zookeys.801.24680

**Published:** 2018-12-03

**Authors:** Catherine Souty-Grosset, Ariel Faberi

**Affiliations:** 1 Université de Poitiers, UMR CNRS 7267, Laboratoire Ecologie et Biologie des Interactions, Equipe Ecologie Evolution Symbiose, 5 rue Albert Turpain, TSA 51106, 86073 Poitiers Cedex 9, France University of Poitiers Poitiers France; 2 Terapéutica Vegetal, Grupo Investigación Zoología Agrícola, Facultad de Ciencias Agrarias, Universidad Nacional de Mar del Plata, Argentina Universidad Nacional de Mar del Plata Mar del Plata Argentina

**Keywords:** agroecosystems, detritivores, ecosystem services, food sources, pests, tillage, woodlice

## Abstract

Terrestrial isopods (approximately 3700 known species in the world) are encountered in temperate and tropical regions, from the seashore to high altitudes and from floodplain forests to deserts. They are known to contribute to soil biodiversity. Environmental factors and anthropogenic actions, particularly land use changes such as primarily agricultural practices, and urbanization affect soil biodiversity and their functions. Human practices, such as soil tillage, pesticide application, chemical pollution, along with soil acidification adversely affect isopod abundance and diversity. It is thus important to recognise the vital contributions of soil biodiversity in support of environmental quality protection through maintaining soil functions and their significance to sustainable land use. This review will also deal with recent studies attempting to evaluate the impact of returning to an environmentally friendly agriculture by restoring refuge habitats such as grass strips, hedges, and woodlands for terrestrial isopods.

## Introduction

Among the most important anthropogenic influences on climate are changes in competing land uses such as agriculture. Global croplands, pastures, and plantations have expanded in recent decades, accompanied by large increases in energy, water consumption, and agrochemical consumption, leading to considerable losses of biodiversity.

Agriculture is a dominant form of land management and agroecosystems cover ca. 40 % of land surface (Millennium Ecosystem Assessment 2005, Power 2010). Agricultural productivity depends on several ecosystem functions, such as decomposition and nutrient cycling by microbes and soil fauna, pollination by animals, biological control of pests. In agroecosystems, soil organisms actively influence soil fertility (Lavelle et al. 1994). The soil fauna mediates a number of essential ecological processes that are vital to the entire ecosystem, such as the degradation of organic matter, cycling of nutrients, sequestration of carbon, and the development and maintenance of soil structure which influences gas and water transportation. Macrofauna such as terrestrial isopods (woodlice) process dead organic matter and facilitate bacterial and fungal decomposition by mechanically breaking up residues and dispersing microbial propagules (Zimmer 2004; Hattenschwiler et al. 2005; Špaldoňová and Frouz 2014). Additionally, by preferentially feeding on certain fungi, isopods alter microbial community composition, and indirectly the fungal feeding invertebrate community (Crowther et al. 2014). As litter transformers, terrestrial isopods can utilize more than 10 % of the annual litter, increasing fourfold the surface available to micro-organisms (Jambu et al. 1987, Mocquard et al. 1988). Moreover, as ecosystem engineers, they contribute to bioturbation of the soil. Bioturbation allows water concentration and thus the formation of moist microenvironments. Feedback mechanisms linked to bioturbation need to be considered at the geomorphic and ecosystem levels in relation to runoff and erosion processes. In *Hemilepistusreaumuri* (Milne-Edwards, 1840), Shachak et al. (1976) showed that by ingestion and defecation of organic matter and inorganic soil particles, this burrowing species could alter the structure of the decomposition substrate and increase the rate of decomposition in deserts. Bioturbation activity leads to a reduction in salt accumulation and to the preservation of a favourable environment for the engineer species (Yair 1995). Isopods also may move litter deeper into the soil (Hassall et al. 1987). One of the most important contributions of invertebrates to soil structure is their feces. The fine structure of soils, and therefore many of its structural features that contribute to soil fertility, is largely determined directly (topsoil) or indirectly (mineral soil) by macrodetritivore fecal dynamics. Consequently, terrestrial isopods are considered as key system regulators of ecosystem functions such as decomposition and nutrient recycling, and affect physical properties of soil.

Terrestrial isopods play a key role in ecosystems influenced by environmental factors, including climate, and so, by global climatic changes (David and Handa 2010, Berg et al. 2010). Terrestrial isopods were first estimated at more than 3,500 species recorded worldwide (Schmalfuss 2003). Recently Sfenthourakis and Taiti (2015) updated the world list of terrestrial isopods containing 3,710 species belonging to 527 genera and 37 families. They are thus studied in several countries and ecosystems. In Europe, the most oniscid species-rich areas are found in the circum-Mediterranean region (Sfenthourakis et al. 2007; Hornung 2011), and a latitudinal gradient in species richness has been shown from the Mediterranean to the northern regions in Europe (Vilisics et al. 2007; Souty-Grosset et al. 2008). Few isopods are included in the IUCN Red List (https://www.iucnredlist.org). Isopods have recently been represented in studies connected to the biodiversity of some protected areas (Szlávecz 1991, Sallai 1993, Almerão et al. 2006, Vilisics et al. 2008, 2011, Messina et al. 2011, 2014) and they are now considered as reliable bioindicators.

The negative impact of land-use on biodiversity in Europe has been documented since the 1990s (Pimentel et al. 1992; Altieri 1999). Decline of grasslands versus crops is threatening diversity of terrestrial isopods (Moss and Hassall 2006). Direct and indirect effects of agricultural management practices negatively affect the abundance and diversity of isopods and in consequence they are generally very low in cultivated plots (Paoletti and Hassall 1999). Direct effects are associated with increases of mortality rates and lowered fecundity due to tillage operations and insecticide application (Fischer et al. 1997). Indirect effects are related to changes in habitat structure and reduced availability of shelter sites and food sources because of herbicide application or burying of plant residues. These herbicides reduce available food and can change soil pH, an important parameter for isopods (van Straalen and Verhoef 1997). It has been observed that the specific diversity and abundance of terrestrial isopods decrease in intensive agricultural systems, with particularly marked differences between organically and conventionally managed plots.

**Figure 1.**
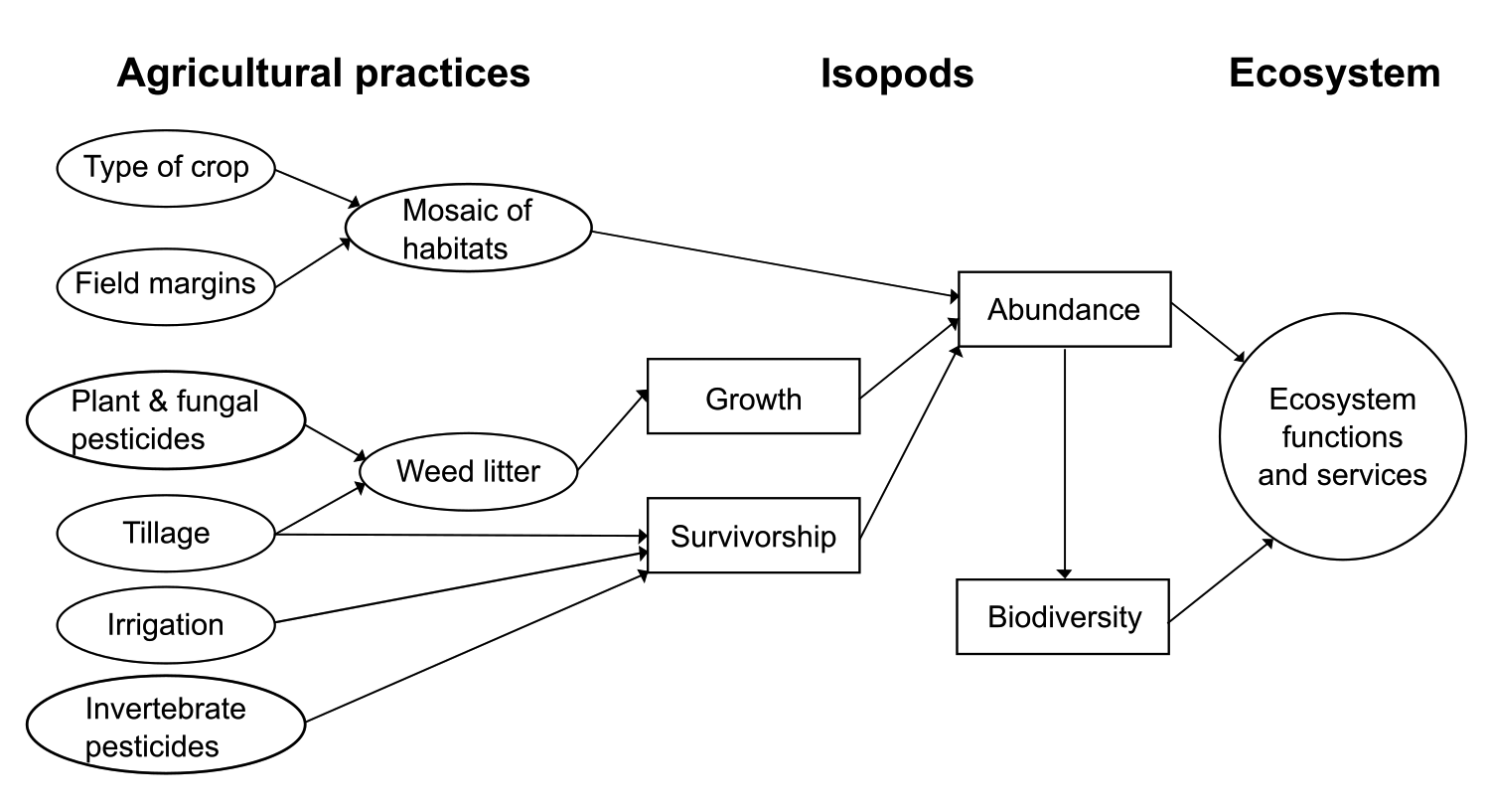
Conceptual diagram illustrating some of the ways in which agricultural practices can influence various aspects of isopod ecology and how these might then potentially impact on ecosystem functions and ecosystem services.

The conceptual diagram (Figure 1) shows how several common agricultural practices can potentially impact the way in which isopods can influence ecosystems. The type of crop, whether pasture, arable or horticultural, helps to determine the mosaic of habitats in the landscape which in turn affects the abundance and distribution of isopods. These are also influenced by the type of field margin (agro-ecological infrastructure), not just by the width of the verge left uncultivated but also whether the margin of the crop itself is managed for wildlife under an agro-environment scheme. Whether the field boundary is a ditch, hedge or fence also determines the habitats available for isopods to use as refugia which can then form a source for future colonization of the rest of the field.

The amount of dicotyledonous plant leaf litter available as high quality food for isopods and hence influencing their growth rates is determined by the type of crop residue and the species composition and biomass of weeds present. These are affected by herbicide applications and possibly indirectly by fungicides but also very much by the type of tillage practiced. As tillage also impacts on the physical structure of the habitat at the soil surface, determining the abiotic favourableness of the habitat, especially its relative humidity which may also be influenced directly by irrigation, both processes potentially impacting on survivorship of isopods and thus on their relative abundance.

The habitat mosaic and population parameters of growth and survivorship interact to determine abundance of different species in different ways, thus influencing biodiversity of the isopod community which, together with the abundance of all the isopod species combined, is instrumental in affecting arable ecosystem functions and hence the level of ecosystem service that they provide. Because of its worldwide distribution, most studies focus on the common pillbug, *Armadillidiumvulgare* (Latreille, 1804).

## Agricultural land-use and diversity of terrestrial isopods

### Preferential habitats of terrestrial isopods

In order to evaluate the impact of agricultural practices on the diversity of terrestrial isopods the preliminary investigations must first include an inventory of species present and their preferred habitat. Paoletti and Hassall (1999) underlined how terrestrial isopods are very widespread, easily identified and form a dominant component of the soil arthropod macrodecomposer community in many temperate habitats (Hassall and Sutton 1977), reaching densities of up to 3000 m^-2^ in calcareous grasslands. Isopods were collected using hand search or pitfall trapping; one of the most commonly used methods of sampling ground-dwelling arthropods (Barber 1931, Duelli et al. 1999, Matalin and Makarov 2011).

They are used as surrogates for natural habitats and grassland biodiversity. They are important primary consumers and are an important food source for other animals, often because of their richness in calcium that is more readily absorbable than in molluscs. The species exhibit a lack of tolerance to low or high values of pH.

**Figure 2.**
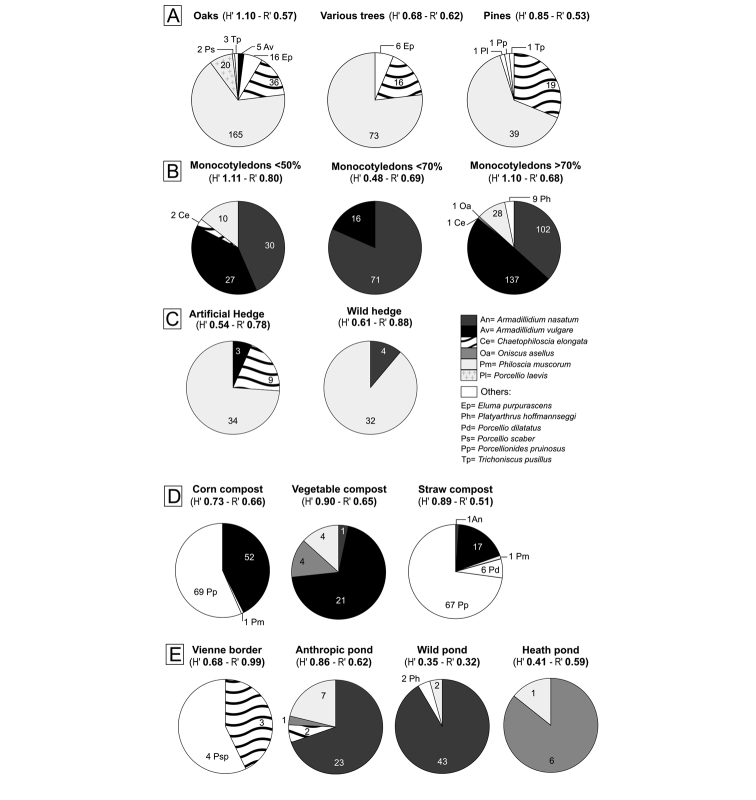
Distribution of isopod species in different types of habitat. **A** Forest **B** Grassland **C** Connections: hedges **D** Compost **E** Humid zones. Shannon (H’) and equitability (R’) indices are written below each graph (from Souty-Grosset et al. 2008).

For example, in Western France, Souty-Grosset et al. (2008) investigated diverse types of habitats in several areas from Poitou-Charentes. The survey was carried out in grasslands (cultivated, temporary or permanent), forests, woods and humid zones, all components of the territory investigated. Humid zones investigated were the margins of ponds and of the Vienne River. Terrestrial isopods were also sampled in connections – either natural or artificial hedges – in order to describe faunal richness in these valuable junctions. Compost heaps and dunghills were also investigated. Sites were randomly prospected with a minimum distance of around 4 km between any two sites. By the end of the survey, 39 different locations were sampled, some of them having several habitats. The study involved 51 habitats. To obtain a correct estimation of isopod diversity, the protocol was simple and reproducible: a hand search method was applied with random sampling for one hour; in order to obtain the same probability of capture, the same three persons were always involved. *Armadillidiumvulgare*, found to be a common species in the region by Legrand (1954), except in forests, was for the first time sampled in oak forests (Figure 2), where the pH is slightly acidic. The species is most prevalent in grasslands rich in monocotyledons, although it has been previously described as preferring dicotyledons (Rushton and Hassall 1983a, b, 1987). No forest harboured *A.nasatum* Budde-Lund, 1885, a species characterized by having a lower tolerance to pH and tannins than *A.vulgare*. Grasslands are the typical habitat of the genus *Armadillidium* by offering suitable pH and humidity. In grasslands, both *A.vulgare* and *A.nasatum* can be present whether or not monocotyledons are dominant. Only grasslands with around 50 % of monocotyledons exhibited a prevalence of *A.nasatum*. The investigation of field boundaries revealed a possible exclusion between *A.vulgare* and *A.nasatum*: wild hedgerows provide *A.nasatum* with higher humidity than do planted hedges; these latter, composed of regularly spaced shrubs with higher temperature and light, harbour particularly *A.vulgare*. Moreover, *A.vulgare* was found in all types of compost, especially from corn and withstanding high temperatures. *A.vulgare* is absent from damp habitats whereas *A.nasatum* was found near ponds as expected. This species is originally a littoral species which colonized inland habitats by following river valleys (Vandel 1960, 1962).

Less abundant species included *Oniscusasellus* Linnaeus, 1758 and *Porcellionidespruinosus* (Brandt, 1833). *Oniscusasellus* is a common species in forests and is a litter feeding macroarthropod, favouring humidity of the soil (Jambu et al. 1987). As the species has no pseudotracheae, it requires a microhabitat with high relative humidity. Moreover, *O.asellus* has a pH preference of around 5.1 (van Straalen and Verhoef 1997), which explains its occurrence in oak and pine forests. *Oniscusasellus* was also collected near water, where the water availability is high, allowing it to take up water by mouth and anus (Edney and Spencer 1955). Composts were dominated by *P.pruinosus*. This species is capable of adaptation to all types of habitats, except those too cold or too humid (Vandel 1962). However, they were absent in grasslands, field boundaries and pond margins. Grasslands and hedges are not favourable because of the high climatic variation and ponds margins are too humid. Composts provide high temperatures to this polyphagous species, allowing high reproductive activity (Juchault et al. 1985).

By comparison, in the Carei Plain natural reserve of north-western Romania, Ferenti et al. (2012) identified 15 species: *Haplophthalmusmengii* (Zaddach, 1844), *Haplophthalmusdanicus* Budde-Lund, 1880, *Hyloniscusriparius* (C. Koch, 1838), *Hyloniscustranssylvanicus* (Verhoeff, 1901), *Platyarthrushoffmannseggii* (Brandt, 1833), *Cylisticusconvexus* (De Geer, 1778), *Porcellionidespruinosus* (Brandt 1833), *Protracheoniscuspolitus* (C. Koch, 1841), *Trachelipusarcuatus* (Budde-Lund, 1885), *Trachelipusnodulosus* (C. Koch, 1838), *Trachelipusrathkii* (Brandt, 1833), *Porcelliumcollicola* (Verhoeff, 1907), *Porcellioscaber* (Lamarck, 1818), *Armadillidiumvulgare* and *Armadillidiumversicolor* Stein 1859. The diversity of the terrestrial isopods in this protected area was high due to the diversity of habitats. The highest species diversity was found in wetlands, with the lowest in plantations and forests. Sylvan species were also present in the open wetlands. Unlike marshes, sand dunes harboured only anthropophilic and invasive species.

**Figure 3.**
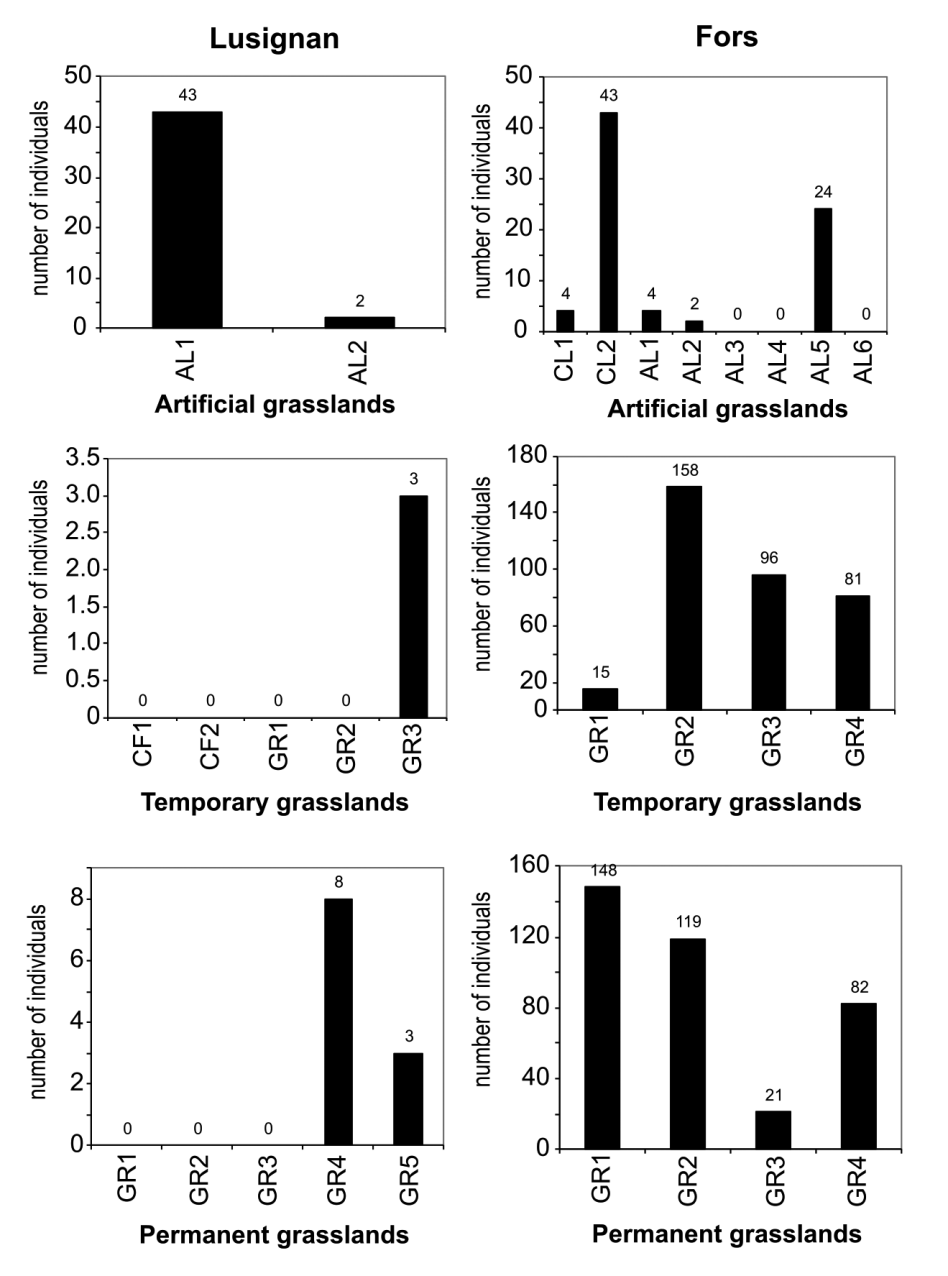
Number of isopods collected by hand-searching in three different types of grasslands in western France. The two sites differ in farming intensity: Lusignan has experienced intensive practices over many years, whereas Fors is in a zone of mixed farming, with a more recent history of intensification. Note the different scales in the y axes (from Souty-Grosset et al. 2005a).

As a result of modern agricultural practices, calcareous grasslands have been declining both in their extent and quality across Europe. As the abundance of terrestrial isopods was described in grasslands Reynolds et al. (2004), Souty-Grosset et al. (2005a) investigated the diversity of isopods in natural and cultivated grasslands of western France, both as grassland detritivores and further considering that their diversity as grassland detritivores could thus be a potential guide to ecosystem activity in natural and cultivated grasslands. Woodlice diversity was studied in different grassland types at two sites: Fors, with mixed farming (crops and livestock), and Lusignan, with intensive farming. Woodlice were collected by hand in plot centres, borders, and field boundaries. Isopod numbers were higher at Fors than at Lusignan. The total numbers of isopods in plots, their borders, and connections (Figure 3) were clearly lower at Lusignan compared to Fors, regardless of the grassland type. Species assemblages were dominated by *Philosciamuscorum* (Scopoli, 1763) at Lusignan whereas this species was less numerous at Fors than *A.vulgare* and *A.nasatum*. These results also differ with grassland type, with high species diversity or number of individuals in temporary and permanent grasslands.

Hedges were important in increasing isopod diversity within plots. The structure of the landscape and its capacity to provide connections between habitats has been found to be important for isopods. The proximity of a suitable habitat for a permanent community of isopods will favour colonization of new habitats. In this study, Souty-Grosset et al. (2005a) showed this influence by the relationship between connections and plots. Thus, hedges are potentially a source of woodlice for grasslands. Therefore, most species present in a plot occur also in the connections. Isopods are highly affected by variations in habitat structure (Davis 1984); while the presence of some species is linked with the degree of openness of the land with some shrubs still present, other species are most common in closed habitats (David et al. 1999). High density of woodlice indicates high habitat quality, as is the case in permanent grasslands. Consequently, some woodlice species may be characteristic of various Atlantic grasslands, and thus are useful as bioindicators of undisturbed and semi-natural conditions. Souty-Grosset et al. (2005b) studied the diversity of woodlice in three types of grasslands: artificial (established for less than 5 years and sown only with leguminous fodder crops), temporary (less than 5 years old, sown with fodder grasses, pure or mixed with leguminous plants) and permanent (sown 6–10 years earlier), in spring, summer, and autumn.

**Figure 4.**
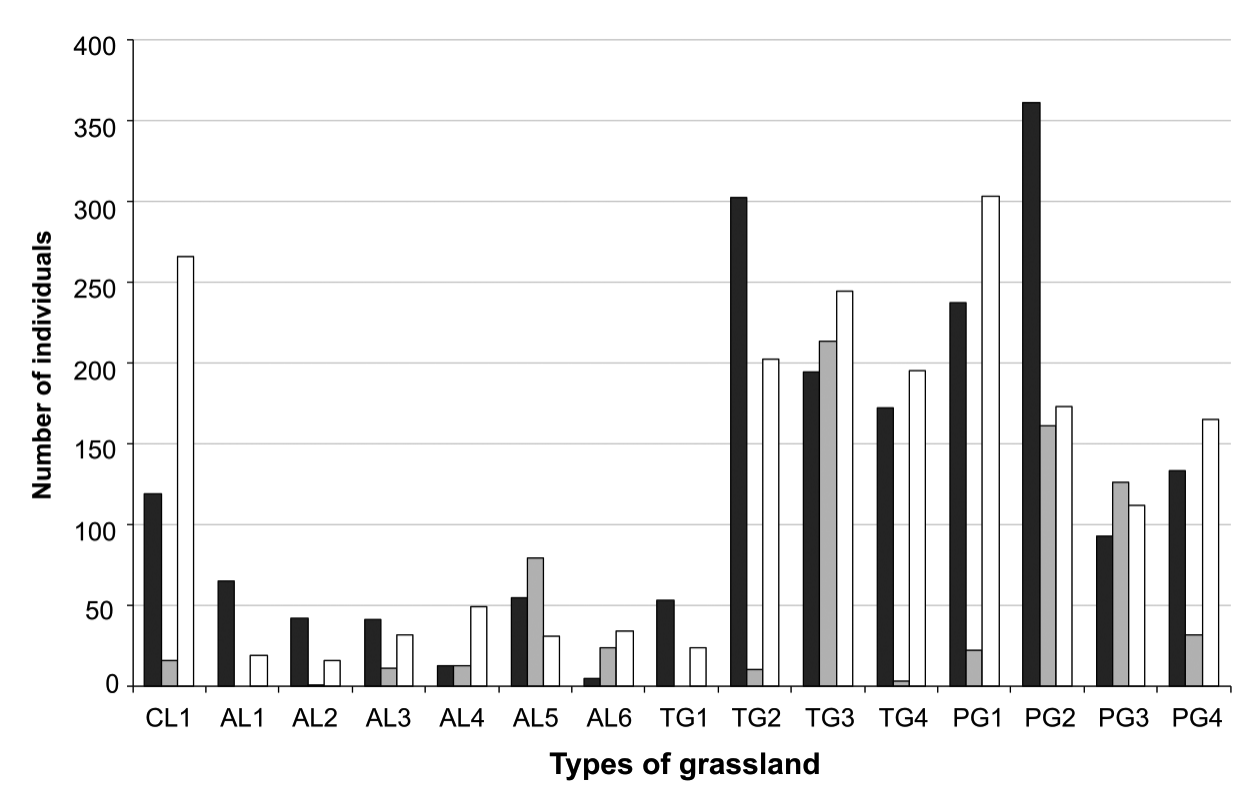
Number of isopods collected at Fors in three seasons: spring (black bars), summer (grey bars) and autumn (white bars). Habitat abbreviations: CL: clover, AL: alfalfa, TG: temporary grasslands less than 5 years old PG: permanent grasslands more than 5 years old (PG) (from Souty-Grosset et al. 2005b).

Relative abundance of isopods was different among habitats and the three sampling periods (Figure 4). Associations were dominated by *A.vulgare*, *A.nasatum*, and *P.muscorum*.

Some species were clearly linked to the degree of openness of the land, agreeing with the conclusions by David et al. (1999). The plot level differences were due to differences in management (cutting or grazing). According to Curry (1994), cutting for silage is a major disturbance for soil arthropods in general. Isopods were most abundant in five years old grasslands. Climatic conditions affect the isopod abundance, and the species dominance may change according to season. In permanent grasslands, populations of *P.muscorum* are most abundant in autumn, *A.vulgare* in summer and *A.nasatum* in spring (personal observation).

Singer et al. (2012) surveyed isopods in the tallgrass prairie ecosystem in Kansas and found the same species as in Western France. Of the four species known in Kansas thus far, all non-native, *A.vulgare* was the most abundant, accounting for 93 % of all individuals. *Armadillidiumnasatum, C. convexus*, and *P.pruinosus* were also found and the authors also reported the first record of *Porcelliolaevis* Latreille, 1804 in Kansas. There was no relationship between isopod abundance and either fire frequency or grazing treatment. Plum (2005), reviewing information mainly from Western and Central Europe, showed that isopods are rare in flooded grasslands. The most frequent species is *Trachelipusrathkii*; all other species occur only occasionally or locally. The most commonly recorded species only occurred in unflooded reference sites (*A.vulgare*, *P.scaber*) or were totally absent (*O.asellus*).

### Diversity of isopods in cultivated plots from a mosaic landscape

**Figure 5.**
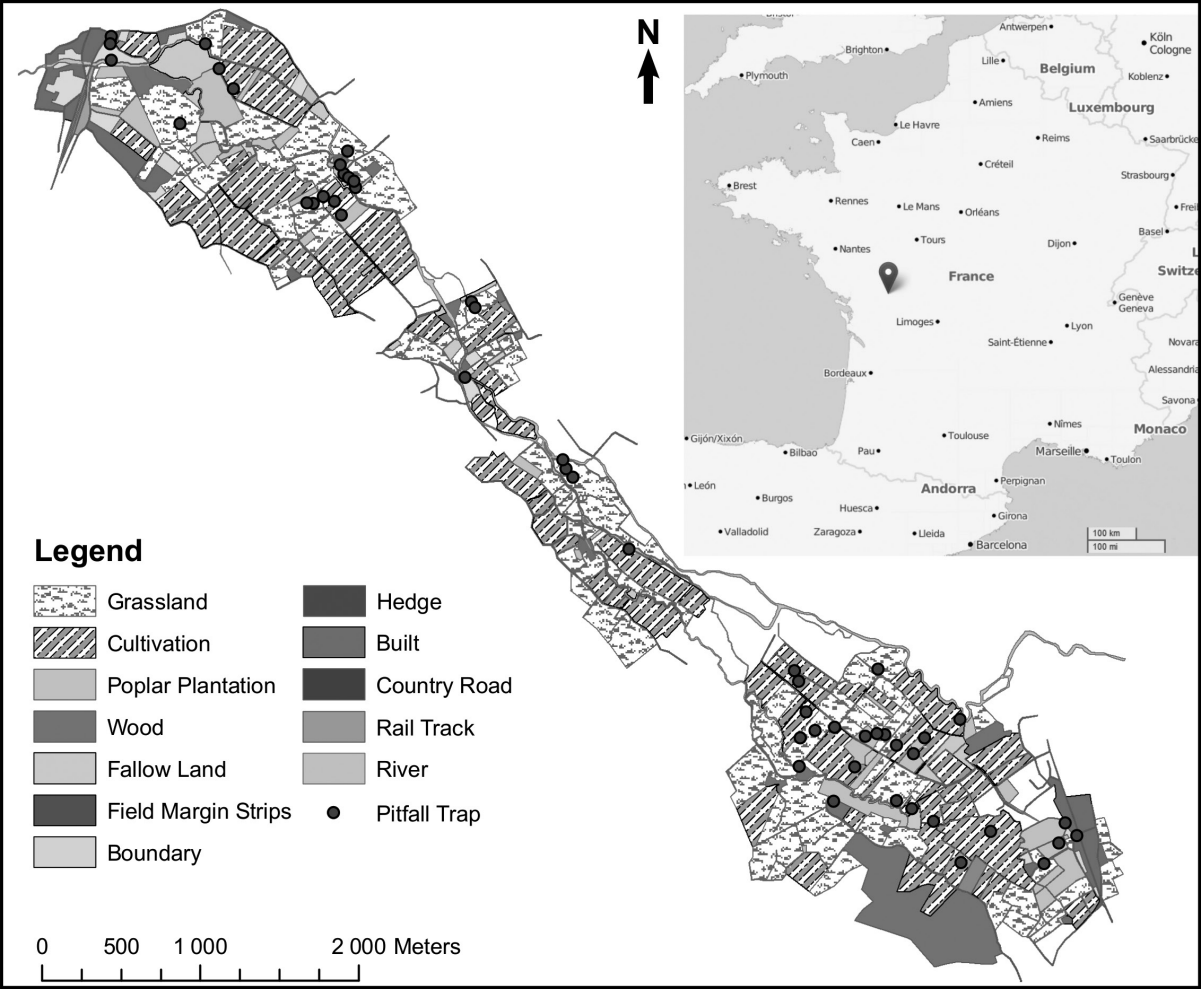
Land cover map with sampling sites in Plaine Mothaise, central-western France. Land cover features of the study site were determined using aerial photographs (Google Earth) and field inspections. Linear characteristics from the landscape were distinguished such as riparian, hedge, continuous and intermittent vegetation. The final categories obtained are Cultivation (crops), Grassland, Poplars, Types of connection, roads and urban. Landscapes were mapped using Arcmap 9.3 (ESRI, 2004) as a main geographical information system. Black dots indicate sites of pitfall trap sampling.

Following the previous study, Souty-Grosset and co-workers (unpublished results) investigated the diversity of terrestrial isopods in a site («Plaine Mothaise»), where changes in agricultural practices led to replacement of more than 25 % of grasslands by crops and poplar plantations within the period 2000–2010 (Figure 5). The approximately 200 ha study area is located between the towns St. Maixent-L’Ecole and La Mothe Saint-Héray (46°22'52"N, 0°06'18"W) in the Poitou Charentes region, central-western France. The localities are connected by the Sèvre Niortaise stream. The area is dominated by croplands (corn), also forest and grassland land uses are represented. This affects habitats, resources and finally the ecosystem services. In order to further integrate restoration management of this area, the impact on terrestrial isopod diversity was first investigated (Table 1).

**Table 1.** Isopod species collected in Plaine Mothaise in different habitat types. Abbreviations: presence of species in grasslands (G), poplars (P), semi-natural habitats (SN) and Crops (C).

Family/Species	Habitat type
** Oniscidae **	
*Oniscusasellus* (Linné, 1758)	GPSN
** Philosciidae **	
*Chaetophilosciaelongata* (Dollfus, 1884)	GPSNC
*Philosciamuscorum* (Scopoli, 1763)	GPSNC
** Platyarthridae **	
*Platyarthrushoffmannseggi* (Brandt, 1833)	P
** Armadillidiidae **	
*Armadillidiumnasatum* (Budde-Lund, 1885)	GPSNC
*Armadillidiumvulgare* (Latreille, 1804)	GPSNC
** Porcellionidae **	
*Porcelliogallicus* (Dollfus, 1904)	GPSN
*Porcelliomonticola* (Lereboullet, 1853)	GPSN
*Porcellioscaber* (Latreille, 1804)	GPSN
*Porcellionidescingendus* (Kinahan, 1857)	SN
** Scleropactidae **	
*Sphaerobathytroparibauti* (Verhoeff, 1908)	PSN
** Trichoniscidae **	
*Haplophthalmusmengei* (Zaddach, 1844)	P
*Oritoniscusflavus* (Budde-Lund, 1906)	GPSN
*Trichoniscoides* sp. (Sars, 1899)	GPSN
*Trichoniscoides* sp. (Sars, 1899)	P

A total of 5 726 isopods were captured representing 15 species and seven families.

The effect of agricultural management on soil arthropod diversity and functioning is often context dependent, e.g. diversity of functionally important taxa such as decomposers may be enhanced by increasing habitat heterogeneity (Diekötter et al. 2010). The role of landscape connectivity in facilitating dispersal between habitat patches has emerged as a key area of research in conservation ecology (Perovic et al. 2010). Arthropod diversity of adjacent agricultural systems can lead to movements of species between them. Thus, even though higher invertebrates are small organisms with specific habitat requirements, at a higher integrative level, diversity evaluation can be based on landscape parameters (Paoletti and Hassall 1999). Several indicators of landscape composition and structure in Plaine Mothaise were calculated. Land cover features of the study site were determined using aerial photographs (Google Earth ^TM^) and field inspections. Linear landscape characteristics such as riparian, hedge, continuous and intermittent vegetation were also distinguished. The following final land use categories were then established: cropland, grassland, forest, connection, roads and urban. Landscapes were mapped using Arcmap 9.3 (ESRI 2004) as a main geographical information system and the database was created.

Table 2 shows the correlation between the degree of response (as measured by the diversity of isopods) and the landscape descriptors. Results show that the riparian vegetation and other connecting elements in the landscape provide higher diversity of terrestrial isopods. Although human modifications of landscape have a negative effect on the diversity, those fragmented landscapes still offering either forest or connections, maintained shelters for isopods.

**Table 2.** Agricultural practices, correlation analyses for landscape metrics and Diversity index of Isopoda in Plaine Mothaise. Field metrics: Total size of site (ha); Length of each type of boundary around focal fields (m). Landscape metrics: Relative area of each land cover type (%) in the landscape; Shannon’s Diversity Index (H = –Rpi * ln(pi)) where Pi = Land cover and I = Total Land cover categories. Shannon’s diversity equals zero when there is only one land cover and increases with both the number of land covers and the evenness of land covers; Shannon’s Evenness Index (HE = H/Hmax = (–Rpi * ln(pi))/ln(M)) of the landscape; N: Numbers of land cover types. Evenness equals one when all land-uses cover the same surface and tends to zero when a land-use dominates the landscape. Landscape structure metrics: mumber of patches: total number of patches in the landscape within each sub site; Mean area-perimeter ratio: sum of the area/perimeter ratio of all patches divided by number of patches in the landscape per sub site; mean patch edge: average amount of edge per patch in the landscape around pairs of fields (m).

Landscape metric	Shannon Index (Isopoda)		
	Pearson Correlation	Sig. (2-tailed)	N
**Num habitats**	0.424	0.477	5
**Num patches**	0.675	0.211	5
**Total area**	-0.795	0.108	5
**Total perimeter**	0.727	0.164	5
**Average area**	-0.744	0.149	5
**Average perimeter**	0.290	0.636	5
**Shannon landscape**	-0.332	0.585	5
**Evenness**	-0.630	0.254	5
% **Cropland**	0.386	0.521	5
% **Refuge**	-0.229	0.711	5
% **Forest**	-0.705	0.184	5
% **Road**	0.277	0.652	5
% **Connection**	0.600	0.285	5
% **Grassland**	-0.083	0.895	5
% **Urban**	-0.392	0.514	5

### Occurrence of Isopoda in cultivated habitats in other parts of the world

In Greece, generally, organic vineyards and maize were the poorest in Isopoda species, while olive groves, both conventional and organic, were the richest (Hadjicharalampous et al. 2002). *Trachelipussquamuliger* (Verhoeff 1907b), the only representative of the Trachelipodidae, was the dominant species in olive groves. *Armadillidiumvulgare*, one of the two Armadillidiidae species found in the studied fields, was the second most abundant species, especially in organic olive groves (Hadjicharalampous et al. 2002).

Studies in other continents show that a drought-tolerant species, such as *A.vulgare*, could have colonised croplands from field margins and boundaries: In Argentina, since the late 1990s *A.vulgare* has been an abundant and frequent species in agricultural land colonizing broad areas (Trumper and Linares 1999, Saluso 2004, Faberi et al. 2011, Villarino et al. 2012, Faberi et al. 2014). *Armadillidiumvulgare* is also found in agricultural lands in Illinois and Kansas, USA (Byers et al. 1983; Johnson et al. 2012) and in the Gauteng Province of South Africa (Tribbe and Lube 2010). *Porcellioscaber* and *Balloniscussellowii* Brandt, 1833 are found although less frequently, the latter recorded only in Entre Rios Province, Argentina (Saluso 2004). Other very common species in agricultural lands are *Australiodillobifrons* (Budde-Lund, 1885) in New South Wales, Australia (Paoletti et al. 2008), and *Trachelipusrathkii* in Pennsylvania, USA (Byers and Bierlein 1984), respectively.

### Tillage systems, agro-ecological infrastructure, and isopod populations

In general, untilled agricultural soils are similar to grassland soils since the absence of tillage allows the accumulation of litter on the soil surface, reducing erosion, modifying the soil surface and topsoil environmental characteristics by reducing soil aeration, stronger mechanical resistance to root penetration, smaller soil temperature amplitudes and thus creating a more favourable microhabitat for soil organisms (Hendrix et al. 1990). In Argentina, as in other parts of the world, during the 1970´s, an intensification of agriculture process took place as agriculture has become more profitable than cattle farming (Studdert 2003, Manuel-Navarrete et al. 2005). Increasing cultivation involved aggressive tillage using moldboard and/or disk ploughs (i.e., conventional tillage - CT). Intense use of CT practices accelerates soil erosion and other degradation processes through its impact on the physical, chemical, and biological factors related to soil quality. In response to these problems, farmers have adopted a conservation tillage system such as no-tillage (NT) as a soil-protecting measure (Studdert and Echeverría 2000, García-Préchac et al. 2004). Under NT, litter and soil organic matter tend to concentrate in the upper 5 cm layer of soil (Dominguez et al. 2005). Since the soil is less disturbed NT practice improves soil aggregation (Carter 1992; Beare et al. 1994) decreases litter decomposition rate (Sánchez et al. 1996, Creus et al. 1998) and reduces organic matter loss (García-Préchac et al. 2004), is. Additionally, in NT systems soil erosion by water and wind is reduced (Fabrizzi et al. 2005, Hobbs et al. 2008).

The litter layer under NT systems enhances habitat conditions favourable for isopods. These include reducing soil temperature and moisture extremes and provisioning of food and shelter (Stinner and House 1990, Wolters and Ekschmitt 1997). On the other hand, soil disturbance in CT has direct and indirect effects on isopod populations. Direct effects are related to injury or mortality of individuals and indirect effects are related to habitat destruction (Wallwork 1976, House and Parmelee 1985, Hendrix et al. 1990, Stinner and House 1990, Holland and Reynolds 2003, Estrade et al. 2010, Errouissi et al. 2011).

According to Paoletti and Hassall (1999), when NT is adopted, isopod biomass and diversity increases compared to CT. In Argentina *A.vulgare* individuals are found both in CT and NT fields. However, this species has taken on considerable importance in agricultural land under NT systems (Trumper 2001, Faberi et al. 2011). Its abundance is higher in NT with respect to CT in winter-spring and in summer-autumn seasons (Manetti et al. 2013). In addition, this species represents a high proportion of the total abundance of arthropods in NT systems, reaching up to 45.5%, while under CT systems it represents approximately 1–9 % of the total abundance of macro-arthropod decomposers (Manetti et al. 2013). The other isopod species, *P.scaber* and *B.sellowii* colonizing agricultural land, were always found under NT systems.

In France, agriculture has changed much during the past 50 years, with the transition from small farms to large farms and the overuse of pesticides causing decreases in biodiversity. In Western France, Deschamps et al. (2011) investigated the diversity of isopods with the return of more environmentally friendly agriculture. With this aim, a group of farmers compiled practices about Major Economic Crops. From 2008 to 2010, the relevance of these modifications of practices was tested. A study was initiated in collaboration with 18 farmers located in Poitou-Charentes and Indre aiming to evaluate the impact of changing farming practices on macrofaunal diversity. Terrestrial isopods have been sampled as bioindicators of the quality of agroecosystems. The abundance of terrestrial isopods is the highest in wheat and agro-ecological infrastructures (AEI). *Armadillidiumvulgare* is dominant in wheat, grass strips, and grasslands. *Philosciamuscorum* is dominant in hedges and secondary in woods. *Armadillidiumnasatum* is the most abundant in grassland and generally less often present than *A.vulgare*. The diversity of isopods is also higher in wheat and agro-ecological infrastructures. In AEI, *A.vulgare* made up 99 % of the total sampling in wheat.

Shannon indices and species evenness (Table 3) showed the highest values in grasslands and hedges, secondly in wheat.

**Figure 6.**
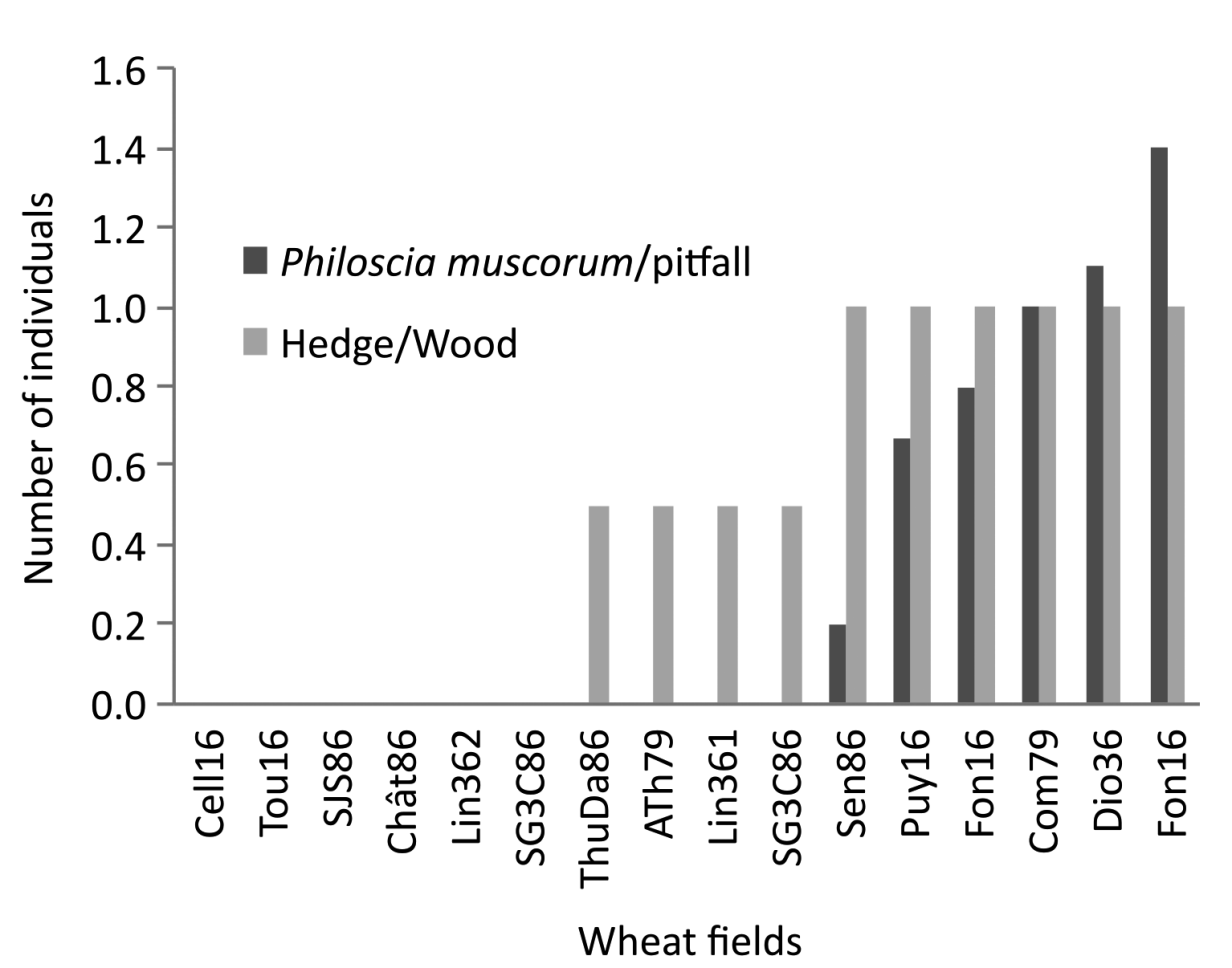
Sampling *Philosciamuscorum* in wheat (pitfall traps). Importance of hedges and woods for inducing the presence of the species in the studied plots. Key: Dark bar: *P.muscorum* in the plot. Light gray bar: *P.muscorum* in the borders of the plot (hedges/wood). Codes were expressed for each plot: the first three letters corresponded to the name of the location, the two following numbers to the French department (16: Charente; 86: Vienne; 79: Deux Sèvres; 36 (1 and 2): Indre).

In the case of wheat plots, *Philosciamuscorum* is encountered in the cultivated plot when a hedge and /or a border is present on the side of the plot (Figure 6).

The size of the plots also affects the number of *Philosciamuscorum* (R² = 0.2954, 29 % of the presence of *P.muscorum* in cultivated plots is explained by the size of the plots). The presence of *P.muscorum* in wheat is correlated both with the presence of agro-ecological infrastructures bordering the plot (the higher the numbers in the hedge, the higher the numbers in the plot) and with the size of the plot (the smaller the plot, more species in the center of the plot are from a nearby field margin).

**Table 3.** Diversity of isopods in plots, hedges and woodland. Shannon indices (H) and species evenness (EN) according to the different types of cultivations and agro-ecological infrastructures as hedges and wood.

Isopods	Wheat			Maize/sunflower			Grassland			Hedges			Wood		
	H’	Hmax	EN	H’	Hmax	EN	H’	Hmax	EN	H’	Hmax	EN	H’	Hmax	EN
**Mean**	0.192	0.318	0.198	0.056	0.069	0.0811	0.316	0.448	0.371	0.403	0.645	0.432	0.156	0.311	0.181
**Min**	0	0	0	0	0	0	0	0	0	0	0	0	0	0	0
**Max**	0.926	1.099	1	0.562	0.693	0.811	0.639	1.099	0.902	1.142	1.386	1	0.652	1.099	0.593

This study shows the importance of refuge habitats like grass strips, hedges and woods for terrestrial isopod populations. Results on *A.vulgare* in wheat were analysed according to tillage, size of the plot, and presence of pebbles (Table 4). PCA analyses showed that the more the soil is tilled, the fewer *A.vulgare* are obtained in samples; also the size and the number of pebbles are not related with the abundance of *A.vulgare* (Souty-Grosset, unpublished data).

Isopod abundance and diversity are related to the type of cultivation, the practices (Tillage and use of phytosanitary products (herbicides, nematocides, and fungicides) expressed by TFI i.e., Treatment Frequency Index, calculated also without Herbicides TFIH-), the size of the plot, the presence of agro-ecological infrastructure (AEI) and its quality AEI+) (Table 5).

**Table 4.** Number of isopods in wheat plots according to tillage, presence of pebbles and the size. Key: *A. v.*: *Armadillidiumvulgare*/pitfall; Till: tillage indices (depending upon the number of rotation and depth); Peb: 1: presence; 0: absence of pebbles; Size: plot size (ha). Same abbreviations used for plots than in figure 6.

Field	A.v	Till	Peb	Size (Ha)
**Sen86**	0	3	0	6.0
**Cell16**	0	3	0	4.2
**Puy16**	0	3	0	7.0
**Chât86**	0	4	0	8.7
**Lin36a**	0	3	0	8.0
**Lin36b**	0	2	0	8.1
**Sg3c86a**	0	3	0	7.5
**Sg3c86b**	0	3	0	8.0
**Thuda86**	0.2	1	0	5.9
**Ath79**	0.2	3	1	6.2
**Com79**	0.2	3	0	6.7
**Sjs86**	1.6	2	1	7.8
**Fon16**	2.2	0	0	3.9
**Dio36**	5.6	2	1	6.0
**Fon16**	162	0	0	2.2
**Tou16**	223.4	0	1	7.5

**Table 5.** Impact of agricultural practices and landscape on the abundance and diversity of terrestrial isopods. (TFI: Treatment Frequency Index: TFIH: Index calculated without herbicides; AEI: agro-ecological infrastructures ; AEI+: agro-ecological infrastructures of good quality): + low significant impact: ++ significant impact; +++ high significant impact).

Isopods	Agricultural practices			Landscape		
	Tillage	TFI	TFIH-	Plot size	AEI	AEI+
**Abundance**	+++	++	+	+++	+	++
**Diversity**	++	+	++	+++	++	++

## Terrestrial isopods and food sources in agricultural systems

While the effects of isopods on decomposition processes and nutrient cycling are rarely considered in agroecosystems, they are beneficial because they provide ecosystem services, enhancing nutrient cycling by comminuting organic debris and transporting it to moister microsites in the soil (Zimmer 2004). In agroecosystems, these functions are very important to allow the continuation of crop production. In agricultural systems, isopod populations must feed on dead crop residue and weeds (Johnson et al. 2012). Different plant species are cultivated in field crops and these are sown and harvested in different seasons through the year. Then their residues dramatically alter the resource input into the agroecosystem. Consequently, isopods are exposed to food sources of different quality and quantity that is also changing temporally.

As an example of agricultural systems in Argentina, crop rotation principally includes wheat, maize, sunflower and soybean crops. When these crops are harvested, different amounts of residues are left in the field, with highest amounts of residues from wheat and maize (7,500 and 6,000 kg ha^-1^ of dry matter), medium amounts from soybean (3,000 kg ha^-1^ of dry matter) and the lowest amount from sunflower (2,000 kg ha^-1^ of dry matter) (Dominguez et al. 2005). Correspondingly, the wheat litter layer provides 100 % of soil cover, maize provides >90 % and both soybean and sunflower provide 65–80 % of soil cover. At the same time, those crops have different chemical properties, such as e.g. the C:N ratio, therefore different degradation rates. Decomposition is slower in residues of wheat and maize with a high C:N ratio) than in residues of soybean and sunflower with low C:N ratio (Sánchez et al. 1996). The monocotyledonous maize and wheat crop residues have high C:N ratios (63 and 60, respectively) while the dicotyledonous soybean and sunflower crops have lower C:N ratios (37 and 45, respectively) (Dominguez et al. 2005). The amounts of residues and soil cover thus decrease faster throughout the decomposition period with residues of soybean and sunflower than of wheat and maize. According to Johnson et al. (2012) the reduction of maize residue levels had minimal impact on *A.vulgare* numbers. The density of *A.vulgare* was similar between fields with a litter layer of wheat and soybean. However, when the amount of soybean residues decreases to 1600 kg ha^-1^ of dry matter, the density of *A.vulgare* was severely reduced (Faberi 2010). In NT systems, the litter layer is a very important component to ensure isopod development, but if this is in low quantity it can adversely affect the stability of isopod populations in the agroecosystem.

**Figure 7.**
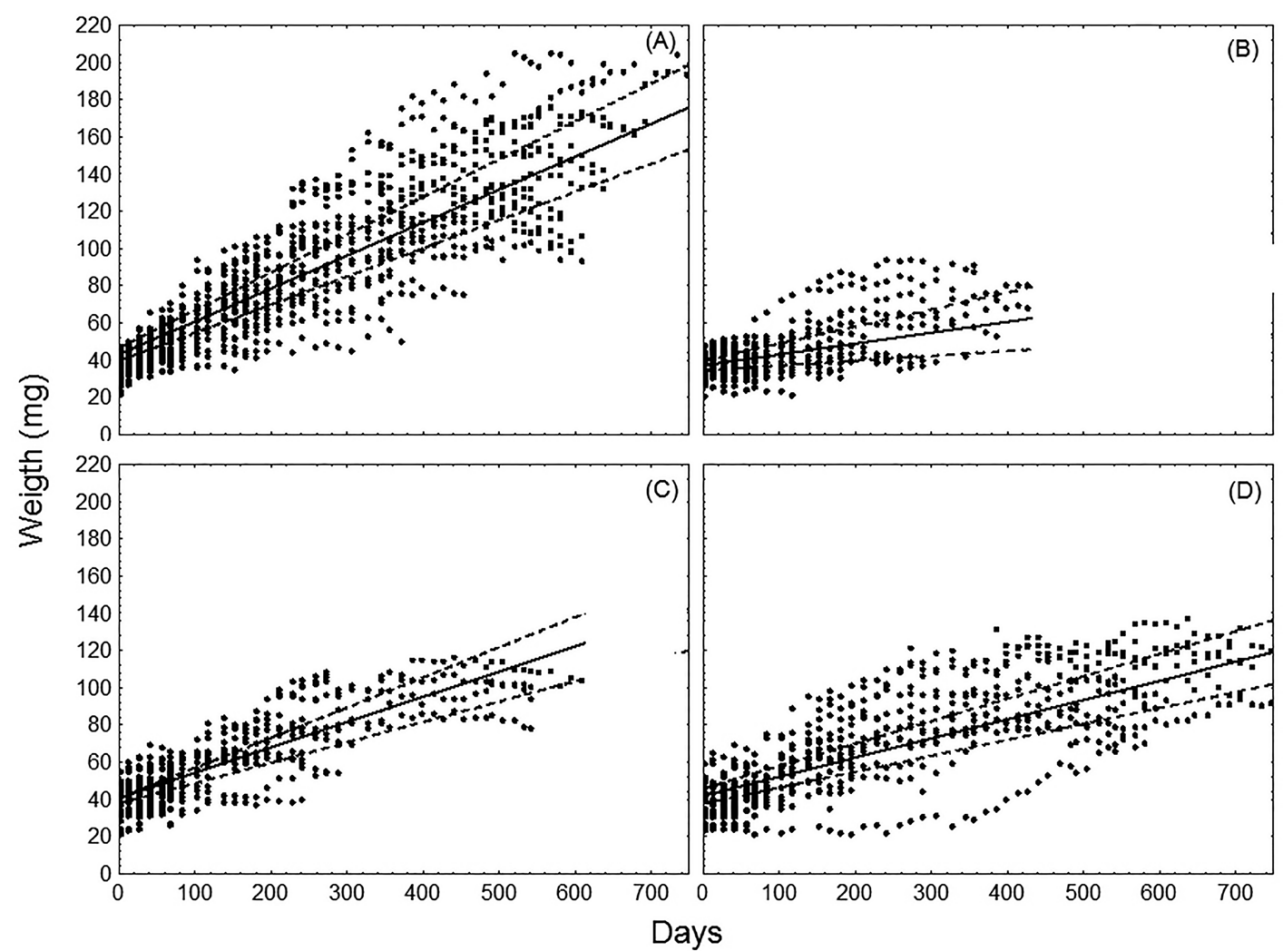
Growth of adult *Armadillidiumvulgare* fed with different types of leaf litter. **A** soybean **B** wheat **C** sunflower **D** pasture. Solid line: Linear growth model, d dashed lines: 95% confidence interval of the model (from Faberi et al. 2011).

**Figure 8.**
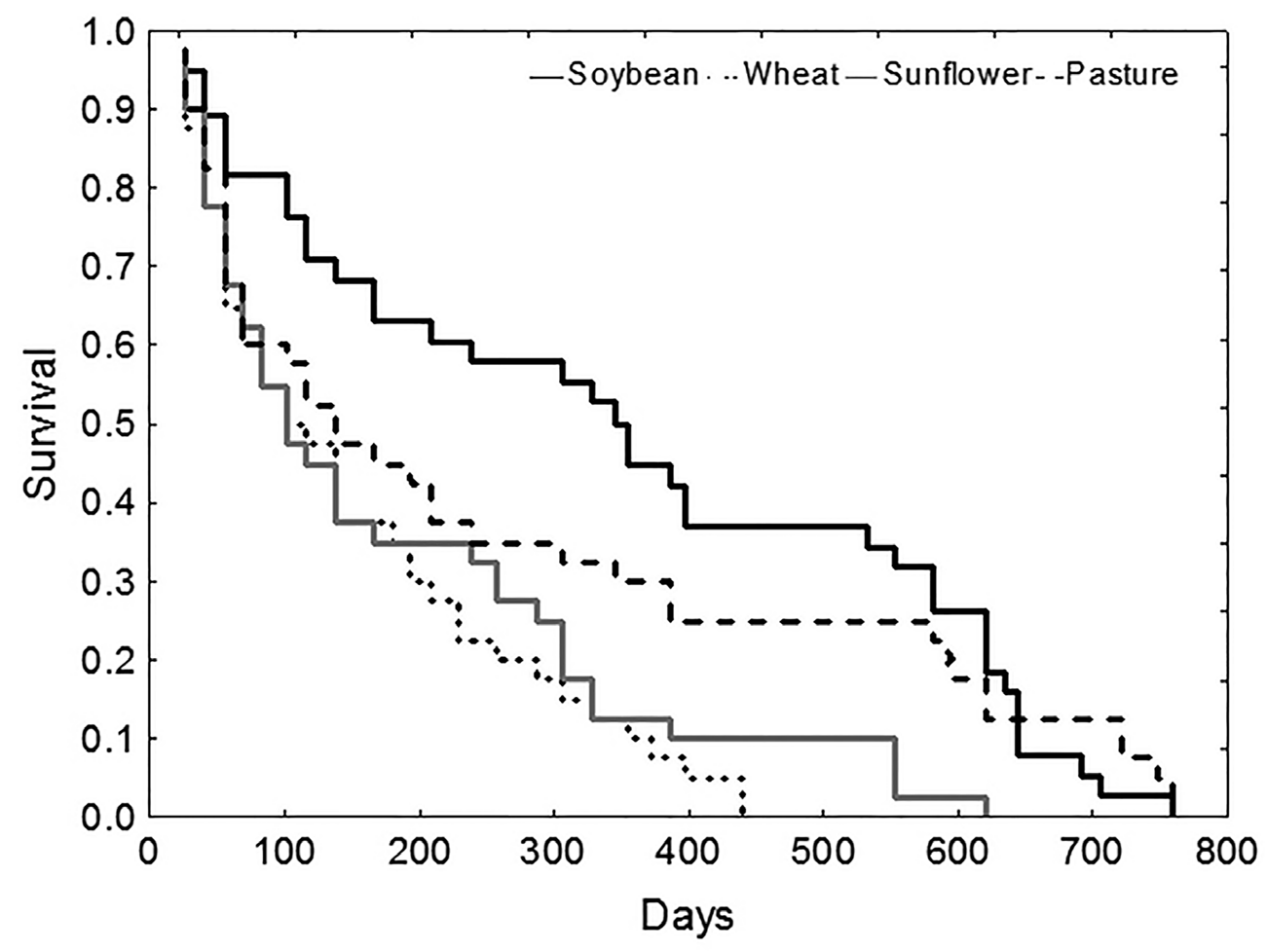
Mortality (Kaplan-Meier method) of adult *Armadillidiumvulgare* feeding on different types of leaf litter: soybean, wheat, sunflower, and pasture during development (from Faberi et al. 2011).

Additionally, the C:N ratio of residues is related to their quality as a food source. Food quality is known to influence the biology of isopods. In general, growth rate and survival are higher when they feed on dicotyledonous leaves than on monocotyledonous leaves (Rushton and Hassall 1983a, 1987, Hassall and Dangerfield 1990, Faberi et al. 2011). The same trend was observed with diets with higher N content as in diets with lower N content (Lardies et al. 2004). Both adult and juvenile *A.vulgare* responded to differences in food quality: in a laboratory experiment; growth rate and survival was higher on soybean residues than on wheat residues (Faberi et al. 2011) (Figures 7, 8). In agroecosystems with a litter layer of soybean it is expected that *A.vulgare* populations have a better habitat from the nutritional point of view.

### Live plant materials as food source: herbivore isopods

Isopods are omnivorous and they have a tendency to shift their food source (Warburg 1993). The capacity of isopods to switch between feeding on dead and green living tissues, i.e., herbivorous feeding, in the field has been demonstrated for several decades. Different isopod species have been observed feeding on green living tissues of several plants even if they have another food source as a choice (Paris and Sikora 1965, Byers et al. 1983, Szlávecz and Maiorana 1990, Hopkin 1991, Benetti et al. 2002; Morisawa et al. 2002; Paoletti et al. 2008; Farmer and Dubugnon 2009; Tierranegra-Garcia et al. 2010, Faberi et al. 2011, Miller 2011, Johnson et al. 2012, Faberi et al. 2014, 2017). In addition, more recently isopods have been reported to exhibit granivory, i.e., feeding on seeds of weeds and crop plants, of some typical plants in agroecosystems (Saska 2008; Koprdová et al. 2012, Salvio et al. 2012).

The preference for or selection of green tissues over decayed leaf litter can be related to the higher N content of green tissues (Szlávecz and Maiorana 1990), or the level or lack of chemical anti-herbivore defense compounds on living plants, e.g. the jasmonate signal pathway (Farmer and Dubugnon 2009) and phenolics (Wood et al. 2012). For example, parallel laboratory experiments identified *P.scaber* and *A.vulgare* as being capable of predation on intact plants. Their feeding was strongly facilitated in jasmonate-deficient *Arabidopsis* and rice plants and revealed potentially detritivore-sensitive, jasmonate-protected Achilles’ heels in these architecturally different plants (petioles and inflorescence stems in *Arabidopsis*, and lower stem and mesocotyl in rice). The work addresses the question of what stops both species from attacking living plants and provides evidence that it is, in part, the jasmonate signal pathway. Additionally, when isopod populations increase in agroecosystems intra-specific competition among isopods is induced and in consequence may result in consumption of live plant material as is observed in *A.vulgare* (Paris and Sikora 1965, Trumper 2001, Saska 2008, Johnson et al. 2012, Faberi et al. 2014) and in *Australiodillobifrons* (Paoletti et al. 2008).

Isopod population outbreaks and their diet switching between dead and live plant material have two possible consequences in agroecosystems. On the one hand, Saska (2008) and Koprdová et al. (2012) suggested that the predation of isopods on weed seeds and seedlings may contribute to biological control of weeds. On the other hand, at really high abundances, isopods themselves can become crop pests.

## Isopods as crop pests

There are reports on terrestrial isopods as a crop pest over several years (Paris 1963, Byers et al. 1983). Commonly synanthropic species have been reported damaging young buds of fruits, vegetables, and flowers. Hopkin (1991) noted that isopods cause significant economic horticultural damage to greenhouse crops. Losses up to 80% have been reported in vegetable crops such as lettuce, tomato, beans, and pepper (Benetti et al. 2002, Morisawa et al. 2002, Whitworth et al. 2008, Tierranegra-Garcia et al. 2011, Miller 2011).

Until the end of the 20^th^ century the general belief was that terrestrial isopods play a beneficial role in agroecosystems, and that their impact as possible pests is limited (Paoletti and Hassall 1999). While there were few documented cases of isopods as pests in field crops, they were seen consuming seedlings only in particular situations in which no alternative food was available (Byers et al. 1983, Wolters and Ekschmitt 1997).

**Figure 9.**
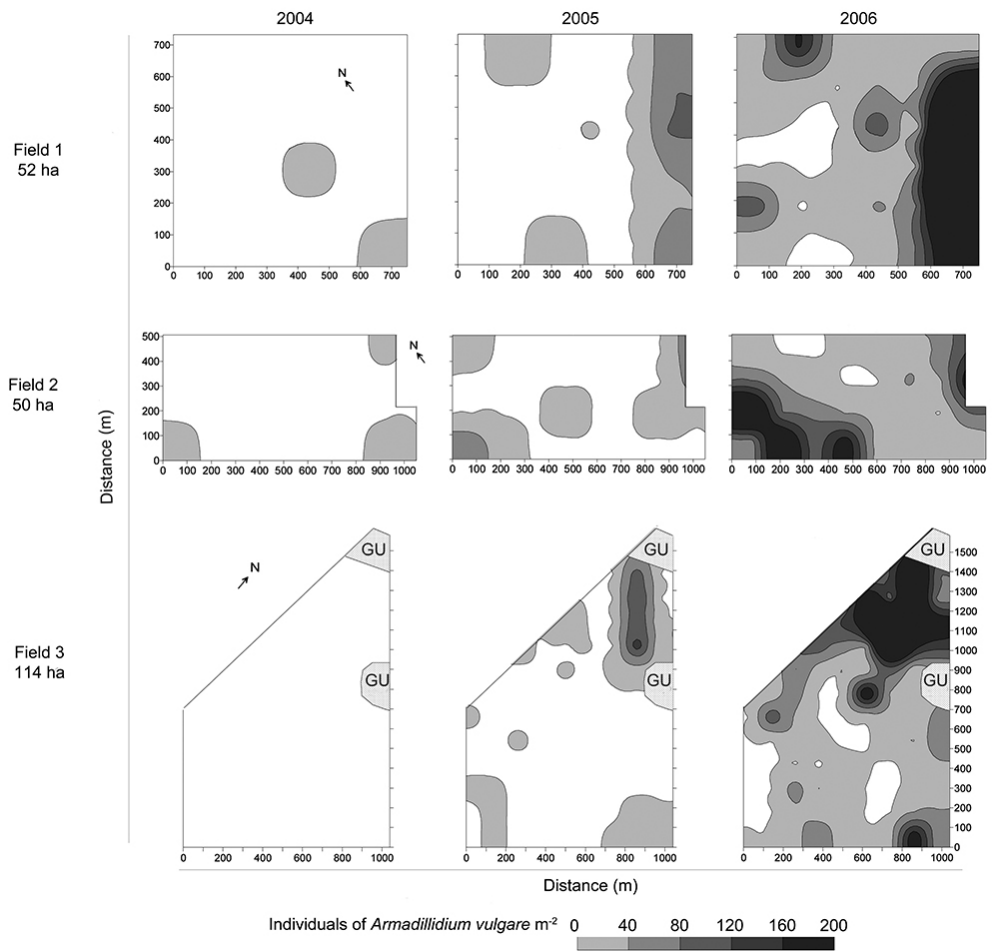
Density of *Armadillidiumvulgare* during 3 years in the same field plots. Samples were collected in October of each year before the sowing of summer crops (from Larsen et al. 2007). Abbreviation: GU: ground under.

At the beginning of the 21^st^ century, as a consequence of the adoption and increased utilization of conservation tillage the number of reports in isopods as pests increased. The first cases were reported in Argentina, where *A.vulgare* was found damaging seeds and seedlings of soybean, and they were named “emerging pests” (Trumper 2001). Then, their populations became pests of sunflower and soybean crops (Mastronardi 2006, Faberi et al. 2014, 2017). This species can quickly colonize field plots within a few years (Figure 9) and reach pest status (Larsen et al. 2007). In South Africa, *A.vulgare* populations are responsible for the loss of canola seedlings (Tribe and Lubbe 2010). A similar situation has been observed in canola crops in Argentina (Villarino et al. 2012) and in soybean fields in the USA (Johnson et al. 2012). *Australiodillobifrons* populations are a pest of cereal crops in Australia (Paoletti et al. 2008). Today, after more than 10 years’ history of NT, isopods have reached high densities and they are recognized in these places as a key pest of several crops under NT system. Recently in central Kansas observations in soybean under no-tillage management revealed that *A.vulgare* was feeding on succulent stem tissues beneath the cotyledons of seedlings, causing significant stand reductions (Alfaress 2012).

Isopod damage to crops is greatest at the time of sowing and immediately after germination, when plants are most susceptible. Monocotyledonous species such as cereals can sustain a substantial amount of grazing from the ends of the leaves without it significantly reducing yield, because grasses and cereals have basal meristems.

The animals feed both on seeds and seedlings, principally at the hypocotyl level, dramatically reducing plant density. These consumptions are correlated with the density of isopods (Faberi et al. 2014) (Table 6). Sometimes crops need to be re-sown due to the extent of feeding damage. In cereal plants isopods crawl up the plant and feed mainly on the tips of the leaves.

In response to this problem, some management practices, such as residue management, planting date and rate, seed treatment, and chemical control have been tested with different efficacy (Tribe and Lubbe 2010, Johnson et al. 2012, Salvio et al. 2012, Villarino et al. 2012). Chemical control alone or with other management practices, is the most effective way to manage isopod populations. An active ingredient (i.e., Carbaryl) is formulated as bait pellets and acts as a neurological poison. The final objective is maintaining the pest population below the level of economic loss, which allows maintaining the plant densities of crops (Faberi et al. 2014, 2017).

**Table 6.** *Armadillidiumvulgare* damage to plants. Pearson correlation coefficient between variables: severe injury in the hypocotyl (SIH), number of plant m^-2^ (NP), yield (Y) and *Armadillidiumvulgare* density (AvD). * (p < 0.05), ** (p < 0.01).

	Agricultural cycle (years)								
	1			2			3		
	Severe injury in the hypocotyl	Number of plant m^-2^	Yield	Severe injury in the hypocotyl	Number of plant m^-2^	Yield	Severe injury in the hypocotyl	Number of plant m^-2^	Yield
**NP**	-0.77 **			-0.94 **			-0.94 **		
**Yield**	-0.61 **	0.68 **		-0.38 *	0.34		-0.88 **	0.81 **	
**Isopod density**	0.54 **	-0.57 **	-0.53 **	0.50 **	-0.57 **	-0.25	0.67 **	-0.60 **	-0.62 **

## Terrestrial isopod diversity related to irrigation

To evaluate the effects of irrigation on terrestrial isopod assemblages Fraj et al. (2010) studied species richness, diversity and relative abundance under three types of irrigation (drip, surface mode and sprinkler) in nine types of cultivation: market gardening (artichoke, tomato and melon); vegetable crop (alfalfa, sorghum and maize) and fruit-trees (apple, pear and olive) in the Majerda low plain in north-east Tunisia. Seven species of terrestrial isopods were identified, with highest richness in sprinkler irrigation. The mean relative abundance of *Porcelliolaevis* was high when the surface mode of irrigation was used. *Porcelliovariabilis* Lucas, 1849 and *Porcellionidessexfasciatus* (Budde-Lund, 1885) showed the highest relative abundance under sprinkler and drip irrigation, respectively. Both Shannon’s diversity and equitability indices were higher with the sprinkler mode. Species richness was higher in the alfalfa and maize cultivation, while mean isopod diversity was higher in the sorghum cultivation. The mode of irrigation has an effect on the abundance and distribution of woodlice at different types of cultivation. The sprinkler irrigation appears to be a good system for isopod diversity conservation. The difference in species richness related to the mode of irrigation could be explained by the fact that, in drip and surface systems, soil moisture exceeded the optimum survival of terrestrial isopods (80% of relative humidity). Thus, sprinkler irrigation may be effectively considered the best for maintaining a good isopod diversity.

## Ecosystem services, conservation issues in agroecosystems

Isopods provide important ecosystem services, such as the decomposition of leaf litter. Usually isopods are rarely considered in agricultural studies and most of the reports are from habitats adjacent to these lands (Wolters and Ekschmitt 1996). During the last hundred years, a drastic decline of natural grassland area all across Europe has been reported (van Dijk 1991). The main reason is that grassland farming is more efficient in cultivated stands than in natural permanent grasslands and urbanization is also having an effect.

Soil biodiversity plays an essential role in the regulation of soil processes that underlie important ecosystem services (Bardgett et al. 2005, Bardgett and Wardle 2010). Traditionally, the value of soil biodiversity in agricultural land has been considered from an economic perspective. However, it is equally important to recognise the vital contributions of soil biodiversity to maintain soil functions which is essential for sustainable land use. Soil organisms are the primary driving agents of nutrient cycle, regulate the dynamics of soil organic matter, soil carbon sequestration, and greenhouse gas emissions, modify soil physical structure, and enhance the level and efficiency of nutrient acquisition by the vegetation (Bardgett and Wardle 2010). Some soil organisms such as terrestrial isopods have been shown to be potentially useful indicators of soil health, because they respond to soil management in time scales (months/years) that are relevant to land management. On the other hand, the level of activity of different species depends on particular management practices, as these affect the micro-environmental conditions, including temperature, moisture, pH, and type of food sources.

## Conclusions

Detritivores, constituting the majority of the soil fauna, i.e., species and functional groups, act in different ways (Zimmer et al. 2004), sometimes with synergistic (positive non-additive) effects (Zimmer et al. 2005). To this end, a diverse soil fauna (Heemsbergen et al. 2004, Hättenschwiler et al. 2005), as well as a diverse vegetation (Zimmer 2002; Hättenschwiler and Gasser 2005), promote decomposition processes and nutrient cycling. On the other hand, soil animals depend on both the composition of the leaf litter (Zimmer and Topp 1997, 2000) and abiotic environmental conditions (Zimmer et al. 2000). Human practices, such as soil tillage and pesticide application, affect soil macrofaunal abundance and biodiversity (Dangerfield 1990, Souty-Grosset et al. 2005a, b, Manetti et al. 2013) and create disequilibrium. Chemical pollution, along with soil acidification, adversely affects soil fauna (Natal da Luz et al. 2004, Zimmer and Topp 1997).

Soil macroinvertebrates have a considerable impact on soil functions important to the restoration process, such as decomposition. Snyder and Hendrix (2008) reviewed how large obligate detritivores (earthworms, millipedes and isopods) have been used to accomplish restoration goals, assess restoration progress, and function as bioindicators. Patterns of detritivore community succession, and how these communities are themselves restored during restoration of perturbed ecosystems, are also discussed. Increasing studies of these taxa are required in ongoing and future restoration projects as well as the outreach activities that should be associated with use of these organisms.

Grassland biodiversity is a function of time among other factors; after disturbance, natural or human-induced, it may take a considerable time for natural communities to re-establish themselves. Plant diversity does not bear a close relationship with faunal diversity, for instance, in fallow land rich in plant species the soil fauna may be poor and unstructured. Isopods as surrogates have considerable potential as indicators of the biodiversity potential of plants in grassland habitats. To develop sampling strategies in order to test community recovery and biodiversity of cultivated grassland plots of different ages in Western France, isopod distribution patterns have been studied (Souty-Grosset et al. 2005a, b). These findings would come into the context of arable systems and help to integrate knowledge on pastoral and arable grasslands and on the influence of management on the grassland fauna (Curry 1994). Following ploughing and reseeding, most soil surface macroinvertebrates must recolonise from adjacent areas. In some long-established grassland plots, species associations within the grassland became similar to those in the boundaries. The permanent ecological corridors such as hedgerows and ditches are important elements of the mosaic of intensively farmed, fine-grained landscapes. Recognising the significance of such landscape features should help in developing guidelines and strategies for conservation management and effective restoration.

Species richness and activity density of woodlice is known to be largely affected by local management and associated habitat characteristics such as soil humidity, pesticide application, or tillage operations (Paoletti and Hassall 1999). The diversity of woodlice was shown to be affected by an interaction of local and regional land use (Dauber et al. 2005). The cover of arable land in the vicinity was found to have no effect on species richness in arable fields, whereas it had a positive effect in grassland and a negative effect in fallow land. As terrestrial isopods play a fundamental role in the agroecosystems, a change in the spatial and temporal structure of Oniscidea communities caused by anthropogenic disturbance may have a cascade effect on ecological cycles, because terrestrial isopods play a fundamental role in nutrient cycling (Magrini et al. 2011).

### New modes of investigation to assess the health of agricultural fields

Rapid biodiversity assessment (RBA): RBA has been proposed by Obrist and Duelli (2010). It is an affordable indicator for monitoring local species richness of arthropods and sustainability of related ecosystem services. The indicator is based on strictly standardised sampling procedures and the identification of parataxonomic units (morphospecies) instead of species identification. Over a period of eight years, annual mean numbers of morphospecies were assessed in Switzerland in 15 agricultural habitats, in 15 managed forests, and in 12 unmanaged habitats ranging from protected lowland wetlands to Alpine meadows. The annual RBA-trend in unmanaged habitats is used for assessing the influence of climate and weather on biodiversity, and as a reference for measuring the relative influences of recent management changes in agriculture and forestry. The average number of morphospecies per sampling station per year depends on temperature, and was only marginally significantly increasing over time in agriculture, but not in forestry or unmanaged areas. Three RBA indices considered to be relevant for maintaining ecosystem services were calculated from the average number of morphospecies per location per year: (1) an indicator for ecological resilience and sustainability (all morphospecies), (2) an indicator for pollinator diversity (taxa with a majority of pollinators) and (3) an indicator for biocontrol diversity (ratio between carnivore and herbivore guilds).

A synthetic index of biological soil quality (IBQS) was developed by Ruiz et al. (2011) studying soil macro-invertebrate community patterns to assess soil quality in 22 sites representing the diversity of agroecosystems encountered in France. Using hierarchical classification, sites could be separated into four homogeneous groups and using the ‘indicator value’ method, 46 indicator taxa characteristic of one or another of these groups were identified. They used a formula that takes into account the abundance of indicator species and their respective indicator values to score soils from 1 to 20. IBQS was able to detect the effects of management practices on soil quality. Soil quality varied from 6 to 20 in forests, 7 to 9 in pastures, and 2 to 9 in crops respectively. Indicator species, such as *T.pusillus* and *O.asellus* had an indicator value of 73 % and 60 %, respectively. Both species are tolerant to soil acidity and their abundances were strongly correlated with soil water-holding capacity (van Straalen and Verhoef 1997).

### Future research

The research must be now conducted in two ways. First, it is necessary to know why isopod population outbreaks occur and what intrinsic or extrinsic factors drive the shift in their feeding behaviour. Second, we need to understand isopod density/crop damage relationships in order to know the lowest population density that each crop can tolerate and then reach agroecological equilibrium. According to Moonen and Barberi (2008), in order to frame the importance of biodiversity in agroecosystems, three main questions were addressed: (1) What does biodiversity mean in natural and agricultural ecosystems; (2) How is the concept of functionality used in relation to biodiversity; and (3) Which biodiversity measures are currently used to express agriculture/biodiversity relationships?

Analysis of the literature was also performed by Moonen and Barberi (2008) and resulted in a framework consisting of three steps. First, the objectives of biodiversity research and policies have to be defined. Three options can be foreseen here: (a) species, community, habitat or overall biodiversity conservation regardless of its functions, (b) biodiversity conservation to attain production and environmental protection services, and (c) use of bio-indicators for agroecosystem monitoring. For example, studying the bioindicator *A.nasatum* at a regional scale, Masson et al. (2014) have developed microsatellite markers as the most efficient markers for studying the influence of landscape features and agricultural practices on genetic structure and demographic history of *A.nasatum* populations. In the second step the appropriate target elements for conservation have to be chosen based on an agroecosystem approach. Finally, the third step involves selection of adequate biodiversity measures of composition, structure and function for each target element. In conclusion, functional biodiversity is important in relation to the provision of specific agroecosystem services. The study of functional biodiversity should start with the definition of agroecosystem functional groups comprising all elements that interact with the desired service, and the subsequent determination of the role of diversity within these functional groups for the fulfilment of the agroecosystem service. Therefore, a more precise definition of ‘functional biodiversity’ was proposed by Moonen and Barberi (2008) as ‘that part of the total biodiversity composed of clusters of elements (at the gene, species or habitat level) providing the same (agro)ecosystem service, that is driven by within-cluster diversity’.
